# Population structure of gut *Escherichia coli* and its role in development of extra-intestinal infections

**Published:** 2010-06

**Authors:** Mohammad Katouli

**Affiliations:** Faculty of Science, Health and Education, University of the Sunshine Coast, Queensland 4556, Australia

**Keywords:** *E.coli*, Urinary tract infection, Gut, Virulence factors

## Abstract

Extra-intestinal pathogenic *Escherichia coli* (ExPEC) strains are divided into uropathogenic *E. coli* (UPEC), strains causing neonatal meningitis and septicaemic *E. coli*. The most common pathotype of ExPEC is found among patients with urinary tract infection (UTI), defined as UPEC. These bacteria are responsible for >90% of cases of UTI and are often found amongst the faecal flora of the same host. *E.coli* strains are classified into four phylogenetic groups, A, B1, B2, and D. Groups A and B1 are commensal strains and carry few virulence-associated genes (VGs) while pathogenic group B2 and D usually possess VGs which enhance colonic persistence and adhesion in the urinary tract (UT). The gastrointestinal (GI) tract is widely accepted as a reservoir for UPEC and is believed that healthy humans have a reservoir of UPEC strains, belonging to phylogenetic group B2, and to a lesser extent, group D. These strains have superior ability to survive and persist in the gut of humans and can spread to cause extra-intestinal infections. ExPEC trains possess a range of VGs which are involved in their pathogenesis. These include adhesins, toxins, iron-acquisition systems (e.g. siderophores), and capsules. Evolutionary influences on the acquisition and main role of VGs amongst *E. coli* are widely debated, with some research holding that the prevalence of strains with VGs increases the likelihood of infections, whereas others believe that VGs provide a selective advantage for infection of extra-intestinal sites. This review is intended to present our existing knowledge and gaps in this area.

**Table:** 

**OVERVIEW OF GASTROINTESTINAL TRACT**	**60**	P-pili	64
**INTESTINAL MICROFLORA**	**60**	Haemolysin	66
*E. coli*	61	Cytotoxic necrotising factor	66
Phylogenetic groups of *E. coli*	61	Siderophores	66
Diarrhoeagenic *E. coli*	61	Capsular polysaccharide	67
Urinary tract infection	62	**SURVIVAL OF UPEC IN THE GUT**	**67**
**BACTERIAL PERSISTEnCE**	**62**	Gut as a source of UTI	68
ExPEC strains causing septicaemia	62	UPEC and septicaemia	68
ExPEC strains causing meningitis	63	**EVOLUTION OF UPEC AND CONCLUDING REMARKS**	**68**
**VIRULENCE FACTORS OF EXPEC**	**63**	**REFERENCES**	68
Type 1 fimbriae	63		

## OVERVIEW OF GASTROINTESTINAL TRACT

The body is protected from the external environment by the intact epithelial layer (1). The GI tract provides a barrier between the external environment and sterile, internal organs, whilst allowing absorption of essential nutrients. The GI system extends from the mouth and oesophagus to the rectum and can be divided into the upper (mouth and stomach) and lower tracts (small and large intestine) (2). Bacteria colonise the GI tract immediately after birth to form a complex milieu constituting hundreds of bacterial species, most of which are residing in the lower intestine. This flora develops through a process of ecological succession and has tremendous role in the state of health and disease. Among the important factors in this process is the influence of the intestinal physiology on the interaction between the microorganisms that contaminate the host, diet regime and food composition, immunological status of the host and the environment (3). The GI tract is composed of four layers: the mucosa, submucosa, muscularis externa and serosa (4, 5). The mucosa is the external layer of the epithelium, which is constantly exposed to bacteria (1, 4, 5).

The intestinal epithelium is more than just a physical barrier protecting internal body sites, with immune interactions to prevent infection and disease from constant exposure to bacteria (6). In the lower GI tract, bacteria must overcome host defences such as peristalsis, lysozyme secretions, intestinal mucus, and gut-associated lymphoid tissue (1, 7). Epithelial cells are protected by a layer of mucus and other nonspecific host defences, as well as a diverse range of mainly commensal bacteria (8). Mucous is secreted by goblet cells and acts as a lubricant for the smooth passage of food and faecal matter along the GI tract. It also helps to trap bacteria, preventing adherence to the GI epithelial cells (1, 6). The lower GI tract has a high rate of cell turnover, constantly shedding epithelial cells and many bacteria which have been able to adhere (6). These host defences help to prevent and decrease bacterial adhesion and colonisation in the lower GI tract.

**Keywords:** *E.coli*, Urinary tract infection, Gut, Virulence factors, Gastro-intestinal (GI).

## INTESTINAL MICROFLORA

The large intestine supports the growth of commensal bacteria known as intestinal microflora. In the large intestine, these bacteria interact with, and colonise the epithelial cells. These bacteria survive within the GI tract, receiving nutrients from the host, whilst providing the host with essential nutrients and benefits (6). Intestinal microflora normally persists for long periods of time and their population size, depending on the species, can vary between 108 and 1012 bacteria per gram of colon contents (9). Major bacterial species/genus that inhabit the large intestine are *Bifiobacterium*, *Bacteroides*, *Lactobacillus*, *Clostridium*, *Fuscobacterium* and *Enterobacteria* (2). It is well established that a stable intestinal microflora can prevent the growth of pathogenic strains which have survived the gastric juice of the upper GI tract and have reached the large intestine (1, 7, 10). Intestinal microflora has an important role in preventing intestinal diseases by competitive inhibition of pathogenic strains, and due to a high number of bacteria and their functions it has been referred to as an organ of the body (3). Intestinal microflora has three main functions and effects on the mucosa: protective, structural, and metabolic (1, 3). It provides a protective function by competition with pathogens for nutrients and receptors within the GI tract. Some strains of this flora, such as lactic acid bacteria, produce bacteriocins and lactic acid which inhibit the growth of other bacterial species (1). The metabolic functions of this flora include the synthesis of essential vitamins such as biotin and folate, and fermentation of non-digestible dietary residues (3). Whilst the GI tract has many protective functions to prevent adherence of pathogenic strains, some functions encourage the growth of bacteria. The mucus layer contains mucins which produce saccharides that are utilised as a source of energy by commensal and pathogenic strains (8).

Many factors can impact homeostasis of intestinal microflora such as changes in diet, physical stressors, and degenerative and infectious diseases (10, 11). Food has been identified as a source for new bacterial species to enter the body and persist as microflora (12). Common foods such as yoghurt contain healthy bacteria known as probiotics, which have a protective role in the gut (13). Bifidobacteria are also common gut species with a protective role however, elderly males and females have shown to have lower populations of these protective bacteria than younger adults (14). Molecular based approaches have identified a shift in dominant microflora species amongst the elderly population towards a greater diversity of bacterial species than younger adults and children (15). Furthermore, fewer species isolated from elderly are able to be cultivated (15). Under certain conditions some of these bacteria are able to cause disease of extra-intestinal sites (3, 15). A common commensal GI tract bacterium responsible for many intestinal and extraintestinal infections is *Escherichia. coli* (16).

### E. coli

*E. coli* is commonly found in the large intestine of humans and other warm-blooded animals (2). These strains can be commensal, existing in a symbiotic state providing resistance against pathogenic organisms, or be pathogenic and cause diseases of intestinal and extra-intestinal sites (6). *E. coli* is found in relatively lower numbers than other major commensal bacteria; however, it is the most common cause of intestinal and extra-intestinal disease (10). The pathogenic strains of *E. coli* may carry several virulence factors directly involved in pathogenesis of these bacteria, although commensal strains may also cause disease in immunocompromised hosts (10, 17).

### Phylogenetic groups of *E. coli*

Commensal and pathogenic *E. coli* can be collectively classified into four different phylogenetic groups; A, B1, B2, and D (18). Phylogenetic groups A and B1 mainly consist of commensal strains found in the large intestine of humans and animals, as well as in environmental samples (19). These strains do not normally carry any known virulence factors (20). In contrast, phylogenetic group B2, and to a lesser extent, group D, consist of pathogenic strains and normally carry virulence-associated genes (VGs) that are mostly associated with extra-intestinal diseases (18, 20). Some reports indicate that strains belonging to phylogenetic groups A and B1 can also cause disease of extra-intestinal sites (19), although this has not been consistently supported (21). A recent phylogenetic group E has also been identified, however it is uncommon, and is not widely used (22, 23). This group shares alleles with many *E. coli* strains and includes the enterohaemorragic strain O157:H7 (22). Pathogenic *E. coli* strains can cause three major types of infections: (i) enteric or diarrhoeagenic disease, (ii) UTI, and (iii) blood infection or sepsis and meningitis (24, 25).

### Diarrhoeagenic *E. coli*

Diarrhoea is more common in developing countries and children under five due to poor sanitation and hygiene (26, 27). Intestinal pathogenic *E. coli* are responsible for majority of these cases worldwide (25). *E. coli* that cause disease of the intestinal tract are referred to as diarrhoeagenic *E. coli* (25). Diarrhoeagenic *E. coli* strains rarely translocate the GI epithelium and their pathogenic affect is mostly restricted to pathophysiological changes of the intestinal epithelial cells (6). The major pathotypes are enterotoxigenic *E. coli* (ETEC), enteropathogenic *E. coli* (EPEC), enteroinvasive *E. coli* (EIEC), enterohaemorrhagic *E. coli* (EHEC), enteroaggregative *E. coli* (EAggEC), and diffusely adherent *E. coli* (DAEC) (25). Since this review focuses on ExPEC strains, the mechanisms by which diarrhoeagenic strains of *E.coli* cause intestinal infection will be briefly discussed here. Unlike commensal *E. coli*, diarrhoeagenic strains carry specific surface adhesins which enhance their ability to colonise the GI tract. Once established in the GI tract, the six pathotypes vary in their disease patterns and intensity (25). ETEC is known as traveller's diarrhoea, and like many other diarrhoeagenic strains, it is associated with poor hygiene. ETEC strains produce two toxins, a cholera like toxin called heat-labile toxin (LT), and a peptide hormone-like toxin called heat-stable toxin (ST) (6, 25). EPEC strains express bundle forming pili (BFP) which are involved in bacterium-to-bacterium adhesion and promotes the formation of bundles. These structural changes cause diarrhoea through the loss of absorptive capacity. EAggEC strains cause persistent diarrhoea in children which is believed to be caused by an unidentified diarrhoeal toxin, EAggEC heat-stable enterotoxin 1 (EAST1) (28). EHEC strains bind to mucosal cells through actin reorganisation as seen with EPEC strains. These strains produce Shiga like toxin, with receptors found on intestinal cells and in the kidney, which can lead to kidney failure known as haemolytic uraemic syndrome (25).

EIEC is the only invasive pathotype, penetrating epithelial cells in an endocytic vesicle. These strains lyse vesicles to multiple within cells and then move through the cytoplasm to adjacent epithelial cells (29). Active invasion of colonic cells causes bloody diarrhoea. The most recent pathotype is DAEC which binds to the small bowel in a diffuse adhesion pattern, however there is limited research on the exact mechanism of action of this pathotype (30).

Other virulent strains of *E. coli* may cause a large range of diseases outside the GI tract (31). These pathotypes are referred to as ExPEC and are divided into UPEC which is the most common pathotype of ExPEC found among patients with UTI, strains causing neonatal meningitis and septicaemic *E. coli* (32).

### Urinary tract infection

UTI is one of the most common diseases worldwide. Although it is one of the easiest treatable diseases, it may cause serious complications in healthy individuals if not optimally treated, with many women experiencing recurrent episodes (32). UTI has been defined as the presence of significant numbers of pathogenic bacteria or organisms in the urinary system, depending on the presence or absence of symptoms while recurrent UTI can be defined as two or more episodes within 6 months or three or more episodes within 12 months (33). UTIs are more common among females, with up to 60% of women having at least one episode in their lifetime (34). The disease is the most frequent hospital-acquired infection, affecting mainly women, children, and the elderly (35). Factors such as shortness of urethra, sexual activity, contraceptives, estrogen deficiency, diabetes, obstructing lesions, and genetic factors such as blood group secretor status increase a woman's likelihood of contracting UTI (33, 36).

The lower GI tract is a common source of UTI causing bacteria, with some strains able to colonise the vagina (37, 40). The UTI-causing strains have certain VGs that distinguish them from other commensal *E.coli* of the gut. These strains often originate from the stool where they enter the UT via colonisation of the vaginal introitus and the periuretheral area (6). Bacteriuria is the presence of these bacteria in the UT when the patient has no physical symptoms normally associated with UTI such as pain, frequency and urgency (24). Under these conditions, *E. coli* strains exist in an asymptomatic carrier state without any obvious symptoms of UTI (41). *E. coli* strains that colonise the UT may ascend towards bladder to cause cystitis, which is usually associated with the classic symptoms of UTI, i.e. pain, frequency, and urgency. UTI can proceed from the bladder, via the ureters to the kidney, to cause pyelonephritis, with the possibility of causing irreversible kidney damage leading to kidney failure and death (42).

UPEC strains have VGs that distinguish them from other strains. Some of these VGs such as adhesins are essential for initiating infection in the host, whereas others (e.g. toxins) are responsible for damaging the host to benefit the bacterium. In UPEC strains adhesion is vitally important to overcome host defences in the UT and resist the flushing effect of the urine (6). UPEC can also carry VGs important for survival in body sites lacking essential metabolites, such as mechanisms for acquiring iron. Although *E. coli* is the most common cause of UTI, responsible for >90% of cases in young adults (37), and >60% of cases in elderly individuals (43), in chronic and complicated UTIs bacterial strains are often mixed (44).

## BACTERIAL PERSISTENCE

Women with UTIs that are not optimally treated, have up to 44% chance of recurrence of infection within 12 months (33). A recent study has found 67% of recurrent UTIs were caused by the same *E. coli* strain (37). This and many other results suggest a reservoir for these strains that enable them to cause recurrent infections (38, 39, 45). The former study has also found that the infecting strains for recurrent UTI to be present in the faecal flora of 78% of women, however the dominance of these clones was not reported (38). Moreno and co workers found 30 out of 42 patients carried a UTI-causing strain as the dominant faecal clone, however persistence of the strain in the GI tract was not measured (38). These findings reinforce the importance of faecal flora as a major reservoir for UTI-causing strains. In the case of recurrent UTI it is also shown that the responsible *E. coli* strains may protect themselves from the effect of antibiotics by hiding in the bladder cells. Using a mouse model of UTI it has been suggested that *E. coli* lie dormant within the bladder mucosa (46).

### ExPEC strains causing septicaemia

Septicaemia is the presence of microorganisms in the blood (6). ExPEC strains are the most common group of microorganisms that cause septicaemia. They may enter the blood through open wounds and burns, or may translocate from the kidney or GI tract, a process called bacterial translocation (47). Severe septicaemia is known as sepsis and is associated with increased morbidity and mortality (48). Septicaemia which has originated from the UT is known as urosepsis (49, 50). From the blood, ExPEC strains can infect distant organs, causing multiple organ failure and shock (51). A recent study investigating the virulence of septicaemic *E. coli* strains has indicated these strains may carry a repertoire of VGs ranging from adhesins to iron acquisition system (52).

### ExPEC strains causing meningitis

Meningitis is inflammation of the protective layers of the brain and spinal cord (53). It is a life threatening infection more commonly associated with neonates, and to a lesser extent, immunocompromised children and adults (54). The diseases now-a-days, is commonly caused by *E. coli*. Infection of the meninges differs from other body site attributable to the necessity for strains to cross endothelial cells which form the blood brain barrier. Meningitis-associated *E. coli* have acquired VGs to cross the blood brain barrier, most commonly K1 capsule polysaccharide and S-fimbriae, which enable *E. coli* to cross via transcytosis (5, 55). K1 capsule also protects the bacterium with serum resistance and antiphagocytic properties (54). *E. coli* strains that cause extra-intestinal infections such as UTI, meningitis and septicaemia have VGs which enable their survival and infection.

## VIRULENCE FACTORS OF EXPEC

There are a number of VGs that help extra intestinal *E. coli* to survive the hostile environment of the GI tract and persist in extra-intestinal sites to cause infection. These include adhesins (e.g. type 1 fimbriae and P-pili), toxins (e.g. haemolysin and cytotoxic necrotizing factor), polysaccharide capsules (e.g. K1 and K5), and siderophores (e.g. iron transport systems aerobactin and novel catecholate siderophore *E. coli*). Many of these VGs are controlled by phasevariable genetic switches that control their expression dependant on the environment conditions such as pH, osmolarity, temperature and amino acid concentration (24, 56). Two of the most studied adhesins amongst UPEC are type 1 fimbriae and P-pili.

### Type 1 fimbriae

Type 1 fimbriae are filamentous organelles that cover the surface of the bacterium (57). These fimbriae are encoded by the *fim* gene cluster, with the structural components of the fimbriae composed of *fimA*, *fimF*, *fimG*, and *fimH*, and the pilus encoded by *fimC* and *fimD* (58). *fimH* is the tip of the pilus which mediates binding to glycoproteins (58, 59). These fimbriae bind to oligosaccharides which are located on the cell membrane of body cells, such as epithelial cells.

Type 1 fimbriae are the most common VG amongst UPEC, and are expressed by more than 80-90% of all *E. coli*, including commensal bacteria and pathogens (59). Due to the frequency of type 1 fimbriae amongst pathogenic and commensal strains, it has been concluded that this fimbriae has no correlation with UTI (24). On the other hand, several studies have shown that type 1 fimbriae are crucial during the first stages of infection by UPEC and mediate binding to urethral epithelial cells (57). Type 1 fimbriae, specifically *fimH*, have also been shown to have a critical role in activation of mast cells in the bladder epithelium, which are an inflammatory cell activated during UTI (57, 60). Activation of mast cells is associated with the release of histamine, which initiates the body's inflammatory response to clear the infection, and is typically associated with the symptoms of UTI such as pain and frequency (60). Type 1 fimbriae lacking *fimH* are unable to invade bladder cells, emphasizing the importance of *fimH* for establishing infection of the UT (61). Tamm-Horsfall protein (THP) coats uroepithelial cells with the main role to bind type 1 fimbriae to prevent bacteria from adhering to epithelial cells (62). Distribution of THP varies throughout the UT which may influence results of different research methodologies (24).

UTI can proceed to the kidneys to cause pyelonephritis. THP is abundant within renal epithelial cells, which binds to UPEC strains expressing type 1 fimbriae, to prevent their adherence in the kidney (62). Although type 1 fimbriae are clearly important for adhesion to the uroepithelium, other VGs are needed for subsequent invasion of epithelial cells and infection. Expression of type 1 fimbriae is coregulated with the expression of P-pili which are associated with with UTI and pyelonephritis. Bacteria are able to switch the production of VGs on or off, depending on the environment and the needs of the bacteria, to create many different phenotypes (56). Expression of the *fim* gene cluster is controlled by *fimS* (63, 64) and is co-regulated with expression of P-pili, with each bacterium expressing only one at a time (63, 64). Studies have shown *papB* (a regulatory protein of P-pili) inhibits the activity of *fimB* and increases *fimE* activity, both work together to inhibit the expression of type 1 fimbriae from being switched into the ‘on’ position, hence inhibiting type 1 fimbriae production (65, 66). Phase-switch ability may account for differential expression of type 1 fimbriae in different body sites. A review article by Johnson (24) has analysed the expression of type 1 fimbriae and reported expression of similar portions in urinary (64%) and faecal strains (60%) with blood isolates having an increased expression (80%). These findings collectively suggest an important role of type 1 fimbriae among septicaemic strains of *E.coli* although their exact mechanism in this process is not known. Much research had been conducted on the prevalence of type 1 fimbriae with conflicting results. Several studies have found high expression of type 1 fimbriae amongst pyelonephritis, cystitis (67), or faecal isolates, and other studies have reported equal expression of this pili among pyelonephritis and cystitis strains (60). These studies varied widely in laboratory culture conditions, age, gender, and geographical location of subjects which are likely factors involved in varied results. The switch-phase variation with P-pili could be responsible for the differential production of these fimbriae amongst cystitis and pyelonephritis strains, and may be responsible for variable expression amongst body sites and studies (67). The role of switch-phase of type 1 fimbriae in septicaemia is not well studied. Amongst *E. coli* strains carrying capsular antigen K1, type 1 fimbriae mutants with phase lock ‘on’ have been found to be more pathogenic during meningitis than those with phase lock ‘off’ (68). Despite this, type 1 fimbriation has been shown to be associated with decreased bacteraemia amongst neonatal rats (69). Variable expression of adhesins and other virulence factors enables bacteria to adjust to different environments encountered during the infection process (56, 64).

Several allelic variations of the *fimH* gene have been identified, with at least three phenotypes having slightly different binding ability (70, 71). Pat least one study has suggested low-binding and high-binding variations of *fimH* to mannose residues (70). In this study approximately 80% of bacteria from the large intestine of healthy humans expressed low-binding to mannose, whereas 70% of bacteria isolated from UTI expressed high-binding to mannose (70). Although type 1 fimbriae are found amongst a majority of commensal and pathogenic *E. coli* strains, allelic variations may exist between these two groups.

Whilst type 1 fimbriae have an important role during colonisation of the UT, their role in colonization of the GI tract is uncertain (31, 43, 72). These fimbriae are frequently found amongst faecal flora and are thought to be important for *E. coli* colonisation of the GI tract (45, 73).

*fimH* is also able to attach to different cell types such as erythrocytes and macrophages, however attachment is thought to occur through different mechanisms than epithelial cells (75). These fimbriae encourage adherence to phagocytes and subsequent phagocytosis (24). Once phagocytosed, type 1 fimbriated bacteria can survive within vacuoles as long as they are unopsonised (76). This remarkable ability helps bacteria to overcome host defences and persist to cause disease.

The GI tract has a range of protective defences, of which sIg A is an important host defence against enteric bacteria (77). GI epithelial cells secrete sIg A in mucous, which bind mannose-containing oligosaccharides and agglutinate bacteria in the gut to prevent epithelial cell adherence and penetration of the intestinal barrier. Mucin and sIgA may contribute to this process by binding type 1 fimbriae and trapping bacteria in mucous (77). Production of a biofilm by commensal bacteria helps prevent pathogenic bacteria from adhering to and translocating the GI epithelial barrier (77). Type 1 fimbriae have also been shown to be important for the formation of intracellular bacterial communities, which have similar functions to biofilms (61).

### P-pili

Another important adhesion for UPEC is pyelonephritis-associated pili, known as P-pili. P-pili are the second most common adhesion in UPEC strains, after type 1 fimbriae (24). These pili are associated with UTI and pyelonephritis, and have been isolated from more than 80% of pyelonephritis causing strains (78). P-pili are assembled by proteins from a *pap* gene cluster composed of subunits *papE*, *papF*, *papG*, *papA*, *papH*, *papC*. Other *pap* genes are important for fimbriae assembly but are not directly related to adhesion, such as *papD* which is responsible for stabilising translocation (6). *papB* is involved in regulating expression of type 1 fimbriae, as previously discussed (56, 64).

P-pili are common VGs of *E. coli* strains belonging to phylogenetic groups B2 and D, and are regularly found among strains that cause UTI (38, 79). While type 1 fimbriae are important for initial infection of the lower UT, P-pili are involved in colonisation of the upper UT (43, 64, 80). Furthermore, recent evidence suggests that type 1 fimbriae and P-pili are inversely regulated, with individual bacteria expressing only one type of fimbriae sequentially (64). Regulation of genes appears to be related to pathogenesis by enabling sequential colonisation of different UT tissues (80, 81). Hence, both fimbriae play an important role in the survival and pathogenesis of UPEC, firstly in colonisation of the GI tract, and then invasion of the UT (38, 43, 73, 74).

P-pili were firstly recognised by the ability to agglutinate human type O erythrocytes without inhibition by mannose, distinguishing it from type 1 fimbriae (6). It has been shown that a common P blood group antigen glycosphingolipid with a lipid moiety and carbohydrate chain to be the receptor for P-pili, α-D-Gal-(1→4)-β-D-Gal (Gal-Gal moiety) (82). This antigen is present in glycoproteins in humans and is found abundantly on the surface of epithelial cells lining the UT (82). The presence of this receptor on epithelial cells of the GI tract has not been fully investigated but it has been shown that resident *E. coli* strains of healthy adults contain a high rate of P-piliated *E. coli* strains (31). Attachment of P-pili to the receptor leads to the release of ceramide, acting as an agonist of Toll-like receptor 4, activating immune cell response (83). Epithelial cell activation leads to the production of cytokines and chemokines, such as interleukin (IL)-6, IL-8 and neutrophils (84). This in turn leads to the development of local inflammation and pain associated with UTI (84). P-pili expression of asymptomatic UTI has been found to be less than cystitis and pyelonephritis causing strains (85). Gal-Gal moiety receptors are found in larger amounts in renal glycolipids than those from shed uroepithelial cells, accounting for P-pili association with pyelonephritis. Receptor density (Gal-Gal moiety) on human uroepithelial cells are equal amongst men and women (24), suggesting other factors are important for increased UTI incidents among women.

Unlike type 1 fimbriae, P-pili do not adhere freely to human polymorphonuclear leukocytes (hPMNLs), given that these cells only produce small amounts of Gal-Gal receptors (83). In strains that also express type 1 fimbriae, P-pili may defend hPMNLs from adhering to and destroying the bacterium (6).

Healthy humans are believed to have a reservoir of ExPEC strains, belonging to phylogenetic group B2, and to a lesser extent, group D, which have superior ability to survive and persist in the gut of humans (86), and can spread to cause disease (87). Interestingly, it has been suggested that P-pili expression enhances colonisation in the GI tract (31, 38, 73, 88). Wold *et al* (31) studied the prevalence of *E. coli* strains carrying this VG among the resident strains of the gut in healthy individuals and found a majority carried P-pili. Based on these results, it has been suggested that P-pili have evolved in *E. coli* strains to promote their persistence in the gut by attaching to Galα1g4Galß-containing receptors on gut epithelial cells (73). Wold *et al* (74) also found type 1 fimbriae bound to colonic cells and to a substance loosely associated to the epithelium, however, P-pili only bound to the loosely associated substance and not the colonic cells (74). Continuous shedding of epithelial cells in the large intestine with Gal-Gal receptors may provide a niche for bacteria containing adhesions to establish colonisation within the gut (74). Gal-Gal binding strength to the UT epithelium is greater than that to colonic epithelial cells, and remains stronger after repeat washes, indicating that P-pili are well adapted for adherence in the UT. The dominance of these clones in the GI tract, and the presence of VGs are contributing factors of UTI (38). This suggests that colonic *E. coli strains*, which are persistent in the GI tract, have mechanisms associated with UTI that may be involved in early colonisation of the urethra (31, 73, 79, 86).

Resident strains of *E. coli* in infants, school girls, and young women commonly belong to the phylogenetic group B2 (79, 86, 90) and express P-pili and type 1 fimbriae more commonly than transient strains (86). For infants (aged 3 days to 12 months) half of these resident commensal B2 strains carried *papC* genes, which is believed to lead to persistence within the GI tract (79, 86). Zhang and co-workers found P-pili amongst young women (aged 18 – 39 years) was strongly associated with phylogenetic groups B2 and D (90). However group B2 had two distinct subgroups with differing levels of pathogenicity. Zhang's findings suggest that healthy adults are capable of carrying B2 strains however with less virulent subclasses than UTI isolates (90). Contrary to these findings, Schlager *et al* (89) found in healthy young girls (aged 3 to 6 years), resident strains with P-pili were not associated with UTI, despite clones of non-dominant strains with P-pili present in the UT. This was based on the findings that dominant clones in the GI tract varied weekly and did not reflect those in the urine, hypothesising that UTI strains are in the gut for only a short period. These results differ from studies of women (15 – 65 years) with dominant faecal clones representing the same urine clone however the study design only included one faecal sample at the time of UTI (38). Further studies have found dominant clones of the faecal flora are more likely to spread to the UT (38, 89, 90). Incidents and pathogenesis of male UTI are not as frequently studied as females (91).

P-pili mediate binding to epithelial layers containing Galα1g4Galß through the use of the adhesion molecule *pap*G located at the tip of the pilus (92, 93). There are three types of *pap*G, each with slightly differing binding ability, these are *pap*G allele I, *pap*G allele II, and *pap*G allele III (92, 93). The prevalence of *pap*G allele I is debated, with some authors concluding it is uncommon and rarely found in humans (93), whilst others report its presence amongst uropathogenic isolates (94). *Pap*G allele II binds to globoside, which is located in the human kidney (95) and is more commonly associated with acute pyelonephritis. Animal model studies have found that *pap*G allele II enhances early colonisation of the kidney, however due to host immune defences, infection was not maintained (93). Cystitis is more commonly associated with *pap*G allele III. The association of *pap*G allele III with cystitis but not pyelonephritis or bacteraemia may be indication that this *pap*G variant is not sufficient for invasion of the bloodstream in non-compromised hosts (93). A study by Otto *et al* indicates that whilst *pap*G allele II was associated with healthy women of all ages, *pap*G allele III was more common in men (96), though this study had a small sample size for male UTI. During bacteraemia, *pap*G allele II has shown to be associated with *E. coli* urosepsis, and *pap*G allele III associated typically with compromised hosts such as those with immunosuppression or UT abnormalities (93). Furthermore, *pap*G allele II has been identified as the predominant variant in *E. coli* bacteraemia (93). Limited information is available on the role of *pap*G variants for intestinal persistence, except that *pap*G class I and II recognise the same receptors in the small intestine and presumably colonic proteins loosely associated with epithelial cells (97).

### Haemolysin

Alpha haemolysin is a pore-forming toxin secreted by pathogenic *E. coli* to lyse erythrocytes and human renal epithelial cells (98). Lyses of erythrocytes releases iron which can be utilised through siderophore systems, hence the production of haemolysin is often regulated by iron availability. Haemolysin is toxic to many cells, leading to inflammation, tissue damage, and disruption of phagocyte function (24, 98). Alpha haemolysin is encoded chromosomally by the gene *hly*A, compared to animals which are encoded in plasmids with differing nucleotide sequences (17). Unlike other toxins, *hly*A gene is expressed without cleavage of peptides or cellular lysis (99). Haemolysin is most active during log phase of growth however activity declines when bacteria reach stationary phase despite continued cell production. This decrease is mainly due to toxic effects of excessive production to *E. coli* (99). Haemolysin production is increased during times of low iron concentration, and decreased in high iron situations (100).

Haemolysin is seen in *E. coli* strains associated with upper UTIs such as pyelonephritis, and is more common amongst invasive uropathogenic strains than healthy faecal isolates (24). It has been reported that an average 12% of faecal *E. coli* produce haemolysin, with similar results supported by other authors (101). Haemolysin producing strains are more prevalent amongst hosts without immune compromising conditions such as renal scarring or pregnancy (24).

### Cytotoxic necrotising factor

Similar to α -haemolysin, cytotoxic necrotising factor 1 (CNF1) is also encoded chromosomally by *cnf1*, with both toxins often co-expressed in UPEC strains (24). CNF1 targets the Rho family of GTP-binding proteins and induces actin cytoskeleton reorganisation, leading to apoptosis, which facilitates bacterial invasion into deeper tissue layers of the UT (102, 103). This process enables bacteria to persist within the UT (104). CNF1 is not as well understood as other UPEC virulence factors. Some authors have reported CNF1 production to promote progressive infection (104, 106), whereas others have suggested no impact within the UT (107). These differences may be attributable to rat models (106), cell lines (105), and human models (107). Haemolysin and CNF1 are strongly associated with phylogenetic group B2, with a stronger association amongst UTI strains than faecal isolates (90, 108, 109).

### Siderophores

Iron is essential for normal bacteria metabolism (110). Free iron in human hosts is limited and not easily accessible to bacteria. Iron in the body is usually found as haemoglobin and heme (111). Bacterial infection can induce an acute-phase response known as hypoferremia to further reduce iron availability (112). During infection, the body reduces the amount of iron available to bacteria, decreasing iron absorption from the gut and storing iron intracellularly (110). The release of transferrin tightly binds free iron, limiting the availability of iron for bacteria. In response to times of limited iron availability, some bacteria release iron chelators known as siderophores, which bind to iron with high affinity (110). There are many types of siderophores which function in different ways, some work to scavenge iron, whilst others compete with host defences to release iron from transferrin and lactoferrin (110). Iron is transported into the cell via outer membrane receptor proteins on the surface of the bacterium specific for each siderophore (110).

In *E. coli*, ferric aerobactin receptor is encoded by the gene *iutA*. Aerobactin has been strongly associated with pyelonephritis, cystitis, and bacteremia as opposed to asymptomatic bacteriuria or faecal strains (24). Aerobactin has been associated with 45 – 78% of UPEC strains (17, 113), with an increased association with bacteremic strains (113). Aerobactin is also associated with persistence in the GI tract, and is more common amongst resident than transient *E. coli* strains hence it may give a competitive advantage for survival and persistence in the GI tract (79, 88). As previously discussed, haemolysin also plays an important role for iron acquisition (114). It has been suggested as an alternative iron acquisition method, with clinical studies finding blood isolates lacking aerobactin were in fact positive for haemolysin production (114). Another identified iron siderophore is *iroN*_E.Coli_. This catecholate siderophore gene is associated with pathogenesis of UPEC and is often coupled with other VGs (115).

### Capsular polysaccharide

Capsules are mainly a polysaccharide structure covering bacteria which acts to protect the bacterium from the host immune system (6). Capsules enhance serum survival, and help to facilitate bacteria to avoid O antigen detection and hence bacterial phagocytosis by hPMNLs (116).

The production of capsules, mainly K1 and K5, are more commonly seen amongst resident strains of the gut, with capsular antigen K5 recognised to enhance gut colonisation (21, 117). Similarly, capsular antigen K1 has been shown to efficiently colonise the large intestine (72), with greater frequency amongst adults than infants. Whilst capsule synthesis is common amongst faecal isolates, synthesis is significantly higher amongst UPEC strains (118). In the UT, capsules help to enhance survival by avoiding phagocytosis (116). *E. coli* strains expressing K1 capsule are the major cause of Gram-negative bacteraemia and meningitis in neonates and have been reported in *E.coli* strains causing cystitis (119).

## SURVIVAL OF UPEC IN THE GUT

Bacterial colonisation of the large intestine is the first stage in the development of enteric, urinary and systemic infections (72). Resident strains are those which are present in the gut from months to years, whereas transient strains are present for a short period of time, usually days to weeks (73, 86). Resident strains are able to adhere and colonise the gut, and mostly constitute the dominant clones in the GI tract. Some studies have found resident strain commonly belong to phylogenetic group B2 and carry a number of VGs (79), whereas others found a majority belonged to commensal phylogenetic groups A and B1 (38, 120).

To survive the human GI tract, bacteria must evade host defences, acquire nutrients for survival, and competitively compete with other microflora (1, 10). Some VGs play a dual role to enhance colonisation of the GI tract and cause infection (38, 121). Bacteria with adhesin molecules specific to gut epithelium have therefore a better chance to colonise the GI tract. P-pili contribute to colonisation of the gut, with resident strains having higher expression than transient strains (31, 73, 74, 79). Studies of healthy adults have found up to 50% of *E. coli* strains carry P-pili (121, 122). However these results vary widely and may be attributable to geographical and lifestyle differences (122). Type 1 fimbriae are also believed to enhance colonisation of *E. coli* in the GI tract (74). Other VGs such as the aerobactin and capsule synthesis may also contribute to persistence in the large intestine (88). Contrary to the production of VGs for enhanced colonisation of the GI tract, Siitonen (101) found a low VGs profile amongst faecal *E. coli* isolates, leading to the conclusion that the gut is not a reservoir for infection. However, this has not been found by other researchers, with many supporting VGs enhancing colonisation of intestinal *E. coli* (73, 74, 79, 93, 121). This inconsistency in results suggests other factors influence the population structure of *E. coli* and their VGs in colonisation of GI tract.

### Gut as a source of uTI

The gut is a common source of bacteria causing UTI. These bacteria can survive and persist in the gut from a few days to many years (10, 79). The finding that *E. coli* strains causing UTIs have been consistently isolated from the faecal flora of the same host (38, 45) strongly supports the gut-origin of UTIs. Intestinal *E. coli* strains causing UTI normally carry VGs necessary for colonisation of the UT and mainly belong to phylogenetic group B2 (90). Jantunen *et al* (50) found *E. coli* strains harbouring *papG* genes were the major facultative flora of the gut in children with urosepsis (aged 1 – 24 months). The way in which these strains come to inhabit the gut is not well established, with ingestion of contaminated poultry proposed as a source of UPEC strains (12, 123). Furthermore, birds have been suggested as reservoir of UPEC although additional reservoirs are likely to exist (124).

### UPEC and septicaemia

In complicated cases of UTI or in compromised hosts, a simple UTI can lead to a serious infection known as septicaemia. From the kidneys, bacteria can enter the blood stream to cause sepsis (125). Urosepsis is the term given to patients with UTI associated sepsis, with the kidney believed to be the source of septicaemia. Studies into the incidents of urosepsis vary, with one study finding 5.9% of sepsis incidents originating from UTI (126), and another reporting a majority at 58.3% (127). Once bacteria have entered the blood they can infect other body organs to cause multiple organ failure, shock, and death (48, 49). Increased bacterial densities also increase the likelihood of a strain colonising the opening of the urethra to cause UTI. In urosepsis, *E. coli* is assumed to move from the UT and kidney to the blood. The gut has also been established as a reservoir of extraintestinal *E. coli*, with patients experiencing UTI, septicaemia, urosepsis or meningitis typically carrying strains with VGs amongst faecal flora (39, 128). Commensal and less virulent UPEC strains have been shown to cause disease in immunodeficient hosts as opposed to uncompromised hosts (17). Therefore, in elderly people, these bacteria have a better ability to translocate to the blood directly from GI tract rather than the UT.

## EVOLUTION OF UPEC AND CONCLUDING REMARKS

It is widely accepted that UPEC strains originate from faecal flora, however whether their pathogenesis is due to higher prevalence within the faecal flora, or the acquisition of VGs, is widely debated (129). The “*prevalence theory”* holds that strains causing infection are predominant within the faeces, increasing the likelihood of these strains colonising the opening of the UT to cause UTI. This is opposed to the “*special pathogenicity theory”* based on the selective advantage of *E. coli* VGs specifically for infection of extra-intestinal sites (129). Irrespective of the evolutionary influences in the development of these VGs, UPEC are well developed for survival in extra-intestinal sites such as the UT. Furthermore, evidences suggest that VGs which contribute to fitness within the UT are likely to enhance GI tract colonisation (31, 85). Intestinal *E. coli* amongst infants, girls, and young women in health and during UTI has been widely studied, however; comparisons between genders and elderly individuals are limited. A review of the existing literature indicates that *E. coli* population structures between young adults and elderly, and genders, could be a predisposing factor to UTI with the GI tract being an established reservoir of infection. Furthermore, *E. coli* populations amongst elderly with weakening immune systems may be an important predisposing factor to disease, especially amongst hospitalised patients. Further researches are needed before these questions can be fully answered.
